# Genetic transformation of lignin degrading fungi facilitated by *Agrobacterium tumefaciens*

**DOI:** 10.1186/1472-6750-10-67

**Published:** 2010-09-14

**Authors:** Krishna K Sharma, Ramesh C Kuhad

**Affiliations:** 1 Lignocellulose Biotechnology Laboratory, Department of Microbiology, University of Delhi South Campus, Benito Juarez Road, New Delhi-110021, India

## Abstract

**Background:**

White-rot fungi are primarily the major degraders of lignin, a major obstacle for commercial exploitation of plant byproducts to produce bioethanol and other industrially important products. However, to improve their efficacy for lignin degradation, it has become necessary to genetically modify these organisms using appropriate vectors. *Agrobacterium tumefaciens*, a soil phytopathogenic bacterium, generally transforms plants by delivering a portion of the resident Ti- plasmid, the T-DNA (transfer DNA). The trans-Kingdom gene transfer is initiated by the activity of Ti-plasmid encoded *vir *(virulence) genes in response to low-molecular-mass phenolic compounds such as acetosyringone. *A. tumefaciens *played a major role in plant genetic engineering and basic research in molecular biology, accounting for nearly 80% of the transgenic plants produced so far. Initially, it was believed that only dicotyledons, gymnosperms and a few monocotyledonous species could be transformed by this bacterium; but recent reports have totally changed this scenario by demonstrating that many 'recalcitrant' species not included in its natural host range can also be transformed, especially filamentous fungi.

**Results:**

This paper describes an efficient and convenient *Agrobacterium*-mediated gene transformation system for successful delivery of T-DNA, carrying the genes coding for β-glucuronidase (*uidA*), green fluorescent protein (*gfp*) and hygromycin phosphotransferase (*hpt*) to the nuclear genome of lignin degrading white-rot fungi such as *Phanerochaete chrysosporium*, *Ganoderma *sp. RCKK-02, *Pycnoporous cinnabarinus*, *Crinipellis *sp. RCK-1, *Pleurotus sajor-caju *and fungal isolate BHR-UDSC without supplementation of acetosyringone. The fungal transformants were confirmed by PCR and Southern hybridization. The expression vector pCAMBIA 1304-RCKK was constructed by the addition of GPD promoter from plasmid p416 to the binary vector backbone pCAMBIA1304, which controls *uidA *and *gfp *gene. Transmission Electron Microscope (TEM) analysis revealed the attachment of bacterial cells to the fungal hyphae. Transformation frequency varied from 50 to 75% depending on the fungal species used in this study. The transformation efficiency was maximum at 20°C whereas no transfer was observed at temperature above 29°C.

**Conclusion:**

These findings provide a rapid and reproducible transformation method without external addition of acetosyringone, which could be useful for improving white-rot fungi for their various biotechnological applications.

## Background

White-rot fungi are primarily the major degraders of lignin. The general attack on lignocellulose by white-rot fungi encompasses a simultaneous decay of polysaccharides and lignin, but preferential degradation of lignin may also occur by selective lignin degraders [1,2]. The technology development based on biological delignification requires the use of a microorganism, which should degrade lignin selectively, rapidly, and exhaustively; process hard wood and soft wood equally and has high growth rate [3]. The microorganism may be expected to have the combination of these properties to determine its efficiency of processing lignocellulosic substrate. The majority of microorganisms lack combination of all properties. However, recombinant DNA technology has led to the emergence of the field of metabolic engineering, the purposeful and directed modification of intracellular metabolism; generate organisms with desirable growth characteristics and cellular properties.

*Agrobacterium tumefaciens *is widely used to transform plant cells [4]. It has the natural ability to transfer a segment of DNA from its Ti plasmid, known as 'T-DNA' into plant or fungal cells so that the T-DNA integrates at random into the nuclear chromosomes [5-7]. As previously reported, *Agrobacterium*-mediated transformation (AMT) also leads to homologous recombination and facilitated gene knock-out. Moreover, AMT improves homologous recombination with appropriate vector constructions [8]. Recently, it has been shown that, under *in-vitro *conditions, the host range of *Agrobacterium *can be extended to non-plant eukaryotes [9-11]. An efficient protocol for AMT of *Aspergillus awamori *conidiospores has been reported by Michielse et. al. [12]. Using diverse *Agrobacterium *strains and isolates, vectors and number of inoculation and selection techniques, transgenics have been produced, which were previously thought to be 'recalcitrant' to *Agrobacterium *mediated gene transformation. The *Agrobacterium *mediated transformation of fungal as well as human cell line (HeLa, HEK293 and neuronal PC12 cells) have been reported [9].

Since last decade, there has been a surge of interest in functional genomics research in filamentous fungi, which has been facilitated by several important advances. In this context, fusing the promoter of gene of interest to a marker gene, such as the gene encoding green fluorescent protein (GFP) from *Aequorea victoria*, is an efficient way to study gene regulation in basidiomycetes (13). There are many possible combinations of transformation systems and functional genomics strategies available, however, all of them are not uniformly successful in filamentous fungi. The *Agrobacterium-*mediated genetic transformation has been proved to be a powerful tool in biotechnology and might become a system of choice for white-rot fungi as well [11]. However, absence of a reliable gene transfer system is the single largest obstacle precluding the use of molecular approaches for the genetic improvement of white-rot fungi [11].

The high transformation frequency, together with the precision and simplicity of T-DNA integration, makes T-DNA a suitable element for genome mutagenesis approaches in white-rot fungi such as gene tagging, promoter entrapment and gene activation, which can also be exploited in different biotechnological applications [11,14]. The genetic manipulation of a microorganism requires the development of plasmid-mediated transformation system that includes: (i) infusion of exogenous DNA into recipient cells; (ii) expression of genes present on the incoming DNA and (iii) stable maintenance and replication of the inserted DNA, leading to expression of the desired phenotypic trait [14]. Here, we demonstrate *Agrobacterium-*mediated transformation of T-DNA carrying genes coding for *uidA*, *gfp *and *hpt *in six white-rot fungi in the absence of acetosyringone.

## Methods

### Organisms

*Phanerochaete chrysosporium*, ATCC 32629 and *Pycnoporous cinnabarinus*, were kind gift from Late Prof. Karl-Erik L. Eriksson, University of Georgia, Athens, USA. *Ganoderma *sp. RCKK-02, *Crinipellis *sp. RCK-1, *Pleurotus sajor-caju *and fungal isolate BHR-UDSC were from our laboratory culture collection. The cultures were grown and maintained on malt extract agar (MEA) containing (g/l): malt extract 20.0, KH_2_PO_4 _0.5, MgSO_4_.7H_2_O 0.5, Ca(NO_3_)_2_.4H_2_O 0.5, pH 5.4 and at 30°C ± 1°C [15].

### Antibiotic selection

Fungal cultures were grown at different concentrations of antibiotics, i.e. 25-150 μmug ml^-1 ^for selection of transformants.

### Plasmid construct and bacterial strains

pCAMBIA1304, which contains a bacterial *hpt *gene driven by Cauliflower Mosaic Virus (CaMV) 35 S promoter and a *gus:gfp *fusion as a reporter gene under the influence of another CaMV 35 S promoter [16] was provided by Centre for Application of Molecular Biology for International Agriculture, Canberra, Australia. *Escherichia coli *strain DH5α and *A. tumefaciens *strain GV3101 were obtained from Prof. Deepak Pental, Department of Genetics, University of Delhi South Campus, New Delhi, India. Vector with glyceraldehyde 3-phosphate dehydrogenase (GPD) fungal promoter, i.e. p416 [13,17] was procured from ATCC vector bank having accession number 87360.

### Vector modification

**Figure 1:**
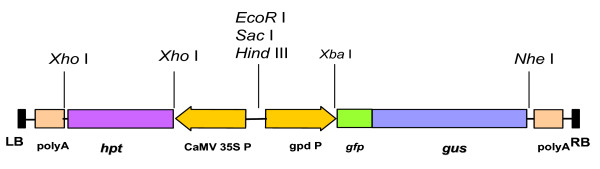
**Modified binary vector pCAMBIA 1304 - RCKK containing GPD promoter**. Hygromycin resistance (*hpt*) gene is under the influence of Cauliflower Mosaic Virus (CaMV) promoter, whereas GFP-GUS genes are expressed using GPD promoter.

The vector pCAMBIA 1304-RCKK was constructed by modifying pCAMBIA 1304, the original binary vector. The *Sac*I and *Xba*I digested GPD promoter, obtained from vector p-416 [17], was inserted to multiple cloning site (MCS) of pCAMBIA 1304. The hygromycin resistance gene was fused under CaMV 35 S promoter [16], whereas GFP - GUS fusion protein was expressed through GPD fungal promoter (Figure 1).

### Fungal transformation

For transformation experiments, *A. tumefaciens *strain GV3101 was grown in 5 ml of yeast extract broth (YEB) containing Kanamycin at concentration of 50 μg/ml for 48 h at 28°C, 250 rpm [11]. One ml of fresh culture was transferred to 100 ml YEB and grown at 28°C, 250 rpm, up to an absorbance of 0.5 at 600 nm.

**Figure 2:**
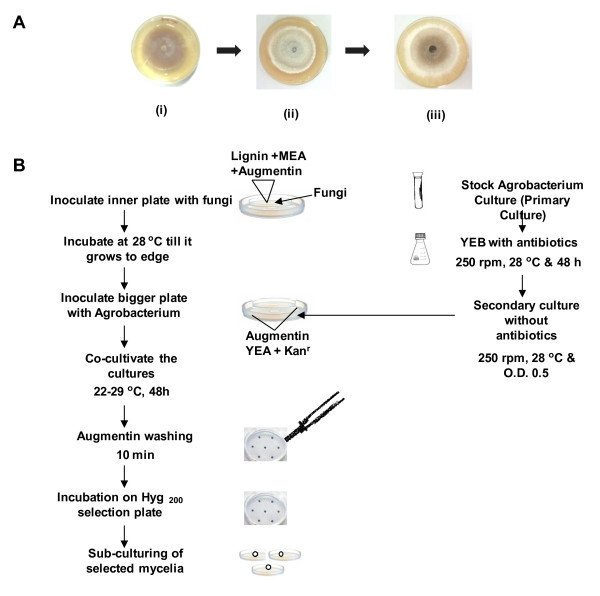
**(A). Stages of co-cultivation of fungus and *Agrobacterium *plates**. Inner plate contains fungal culture and outer contains *Agrobacterium *culture. (i) Innocula on inner plate with lignin (0.1%) (ii) Full-grown fungal mycelia and (iii) Fungal cultures with *Agrobacterium *under co-cultivation conditions. (B). Diagrammatic illustration of *Agrobacterium *mediated transformation of different white-rot fungi.

Two Petri dishes of different diameter were kept in a concentric position, placing smaller one in the centre of bigger Petri dish (Figure 2A). First, the inner Petri dish was poured with 2% MEA supplemented with 0.1% lignin and augmentin (100 μg/ml) and then bigger plate was poured with yeast extract agar (YEA) containing kanamycin at a concentration of 50 μg/ml. Both the plates were poured very carefully so that media do not mix with each other. The MEA plate (inner one) was inoculated at the centre with a fungal disc, incubated at 28°C and it was allowed to grow till the hyphae cross the periphery of the inner plate and move on to the outer plate. Thereafter, *Agrobacterium *culture was plated carefully on the medium in outer plate, which already contains growing mycelia and co-cultivated at temperatures ranging from 20-29°C for 48 h (Table 1 and Figure 2A, B).

**Table 1:** Transformation efficiencies (%) in different fungal species at varying co-cultivation temperatures

Basidomycetous Fungi	Percent transformation efficiency (η)* at different Co-cultivation temperature			
	
	20°C	23°C	26°C	29°C
*Ganoderma *sp. RCKK-02	65	48	32	17
*P. cinnabarinus*	65	45	30	15
*Crinipellis *sp. RCK-1	75	65	48	ND
*Pleurotus sojur-caju*	50	34	45	ND
*P. chrysosporium*	75	63	34	20
Fungal isolate BHR-UDSC	50	30	18	ND

ND- Not Detected

* $\eta =\frac{\text{no}\text{. of transformed pellet positive for GUS}}{\text{no}\text{. of wild type fungal pellets}}\times 100$

After co-cultivation, mycelial discs from peripheral region, where fungal hyphae reached to bacterial lawn, were cut randomly and washed with a concentration of 250 μg/ml augmentin (Medreich Sterilab Limited, India). Thereafter, the mycelial discs were transferred to MEA containing hygromycin at a concentration of 200 μg/ml and augmentin (100 μg/ml), and incubated at 28°C, 144 h. The culture containing Petri plates were sealed with gas-porous tape (Micro-pore, India).

### Screening of transformants

#### Detection of reporter gene expression

GUS histochemical assay was performed as described elsewhere [18]. After co-cultivation of fungal mycelia with *Agrobacterium*, four fungal discs (2 mm dia each) were randomly picked up and grown in malt extract (2%, w/v) at 100 rpm, 30°C for 3 days in the presence of hygromycin at a concentration of 200 μg/ml. Similarly, untransformed control fungal cultures were grown in the absence of hygromycin. This resulted in the development of small pellets and each pellet was considered as an individual transformant. The mycelial samples resistant to hygromycin were stained in X-gluc solution at 37°C overnight. Thereafter, the number of transformants in co-cultivated mycelia were compared with control fungal pellets after GUS staining. Transformation efficiency (η) was calculated as:

$$\eta =\frac{\text{no}\text{. of transformed pellet positive for GUS}}{\text{no}\text{. of wild type fungal pellets}}\times 100$$

For the formation of the GFP chromophore, transgenic mycelia were incubated at 4°C for 2 h [19]. Fluorescent microscopy (Olympus Reflected Fluorescence System, bx 51; Olympus Optical Co., Ltd. Japan) of fungal hyphae was performed using Olympus camera DP70 for the imaging of GFP expression. GFP was detected with an excitation filter BP460-490 and barrier filter BA520IF. Images were captured using Olysia, software imaging application (Olympus Optical Co. Ltd., Japan) and processed electronically using Adobe Photoshop (Adobe Systems Inc., USA).

#### Analysis of T-DNA integration and transgene expression

Genomic DNA from the transformants was isolated as described earlier [20]. PCR analysis was performed with primer pairs 5'-CCGGATCCATGGTAGATCTGACTAG-3' and 5'-GCTTGCATGCTTAGTATAGTTCATCCATGC-3' for *gfp-gus *gene to amplify a 2.5 kb fragment. Genomic DNA digested with *Bam*HI was analysed by Southern hybridization using radiolabelled *hpt *gene probe [21]. All the transformed mycelia were also tested for the possibility of persistent *Agrobacterium *contamination. Fungal mycelia were grown on Luria Broth (LB) medium to screen out the bacterial contaminant. *Bam*HI digested genomic DNA from the transformed lines was analysed by Southern blot hybridization using *Kan^R ^*probe.

### Confirmation of *Agrobacterium*-fungus attachment

Fungal mycelia were harvested from the bacterial-fungal interface agar zone. Thereafter, fungal hyphae were fixed in 2.5% glutaraldehyde and 2% paraformaldehyde, prepared in 0.1 M sodium phosphate buffer (pH 7.4) and subjected to transmission electron microscopy (TEM) (Morgagni 268 D, Transmission Electron Microscopy, Fei, Holand) at Department of Anatomy, All India Institute of Medical Sciences (AIIMS), New Delhi.

### HPLC analysis of phenolics

The fungi were grown on malt extract broth (0.5%) supplemented with micronutrients (g/l), i.e. KH_2_PO_4 _0.5, MgSO_4_.7H_2_O 0.5, Ca(NO_3_)_2_.4H_2_O 0.5 and 1% lignin at pH 5.4 and temperature 30°C under static conditions. The samples were harvested on 10^th ^day and analysed for lignin degraded products. The cell-free culture filtrate was mixed with acetonitrile, vortexed and centrifuged for 10 min to pellet the remaining insoluble material. The supernatant was passed through 0.2 μmum membrane filter. Samples (25 μl each) were applied to a Luna 5 u C18 HPLC column (5 μmum particle size, 250 × 4.6 nm) under isocratic condition of 22% acetonitrile, 78% phosphoric acid from 1% stock. The authentic phenolics standards and lignin samples were analysed for lignin precursors and degraded intermediates by monitoring UV-absorbance at 280 nm, maintaining constant flow rate of 0.5 ml/min (Waters, HPLC, Water Corporation, Milford, MA 01757, USA).

## Results and Discussion

### Transformation and reporter gene expression

Hygromycin at the concentration of 150 μmug ml^-1 ^showed complete inhibition of different lignin degrading fungi, whereas it showed restricted growth at 125 μmug ml^-1^. Other antibiotics tested, i.e. ampicillin, kanamycin, rifampicin, gentamycin, did not show any significant growth inhibition even at 150 μmug ml^-1 ^concentration.

**Figure 3:**
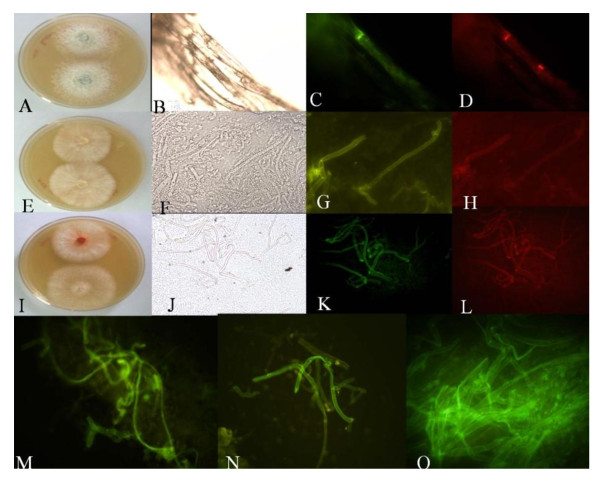
***Agrobacterium *mediated *in-vitro *gene transfer as measured through UV-florescence using different excitation filters (BP460-490) and their hygromycin selection**. (A). *Ganoderma *sp. RCKK-02; (E). *Crinipellis *sp. RCK-1; (I). *P. cinnabarinus *(M). *P. sojur-caju*; (N). *P. chrysosporium*; (O). Fungal isolate BHR-UDSC. (B,F,J) Images under Bright field; (C, D, G, H, K, L) GFP-tagged mycelia using different excitation filters (BP460-490) and barrier filter BA520IF.

The fungal cultures when co-cultured with *A. tumefaciens *harboring *hpt *gene resulted in development of hygromycin-resistant (Hyg^r^) mycelia (Figure 2B). The DNA transfer events were studied by monitoring the transgene associated with T-DNA (*gfp, uidA *and *hpt*) in Hyg^r ^colonies. GUS histochemical assays after co-cultivation showed high frequency of transformation. A high percentage GUS positive cells were detected in the transformed mycelia (about 75% in *Crinipellis *sp. RCK-1 &*P. chrysosporium*), indicating that the cells received T-DNA (Table. 1). A standard transformation system of *P. chrysosporium *was used as a positive control which has been reported earlier [11]. Stable transformants showed the expression of GFP as detected by confocal microscopy (Figure 3). Interestingly Hyg^r ^phenotype was retained even after sub-culturing the transformants in hygromycin- free medium for a period of 2 months.

The transformation results confirm the efficacy of both the promoter, i.e. CaMV 35 S and GPD, in the expression of *hpt *and *uidA *gene, respectively. There are earlier reports of CaMV 35 S promoter in driving the expression of reporter genes in *P. chrysosporium*, *Ganoderma lucidum*, and *Pleurotus citrinopileatus*, the lignin degrading white-rot fungi [11,16]. Moreover, CaMV 35 S promoter used in the transformation studies provides an additional choice in basidiomycetes molecular studies, and allows the construction of shuttle vector system for alternative expression of genes in fungi and plants, which requires only minimal cloning manipulations [16].

The transfer efficiency was maximum at 20°C (Table 1) whereas no transfer was observed at temperature above 29°C. Suppression in transformation at higher temperature could be attributed to loss of activity of *A. tumefaciens *to provide the virulence principle [22,23]. The conformational change in virA, resulting in inactivation of the protein at higher temperature has been reported [24]. Moreover, the sex pili involved in the transfer of T-DNA could be absent or unstable at higher temperature [25]. Our results suggest that co-cultivation at low temperature could significantly increase the transformation frequency.

In the present study, the white-rot fungi when grown in malt extract media supplemented with lignin, were observed to produce lignin degraded products compared to control medium (without lignin). White-rot fungi have been shown to partially mineralize lignin in axenic culture [26]. A large number of phenolic secondary metabolites are also reported in the lignin degradation pathway [26,27]. These phenolic compounds, which are normally involved in lignin biosynthesis, serve as inducers (or co-inducers) of bacterial virulence genes [28]. The *vir*-inducing activities of the lignin precursors have been discussed in terms of the biology of *Agrobacterium *[29,30].

### HPLC analysis of the phenolics

**Figure 4:**
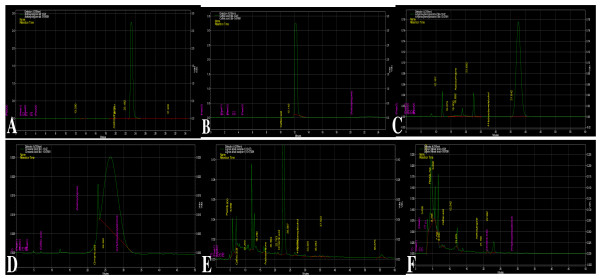
**HPLC profiles of phenolic extracts from lignin (commercial) (E) and wheat bran (F)**. The peak at retention time (RT) 23.19 was identified as acetosyringone. Different compounds of lignin monomer pathways were used as standards, i.e. acetosyringone, RT 23.19 (A); caffeic acid, RT12.142 (B); 4-hydroxybenzylalcohol RT 37.642 (C) and cinnamic acid RT 26.350 (D).

The HPLC profile of the degraded lignin compound confirmed the presence of different intermediates of lignin synthesis pathway. The presence of acetosyringone in fungus-degraded lignin supernatant advocates the transformation of fungal mycelia grown in lignin-supplemented media. In addition to well established *vir*-gene inducer, i.e. acetosyringone, we also detected some additional phenolic compounds like caffeic acid, 4-hydroxybenzyl alcohol and cinnamic acid (Figure 4). HPLC profile also showed few unidentified peaks, which made us to hypothesize that there might be some synergistic or cumulative effect of different phenolic compounds, resulting in high transformation efficiency.

### Molecular analysis of nuclear integration

**Figure 5:**
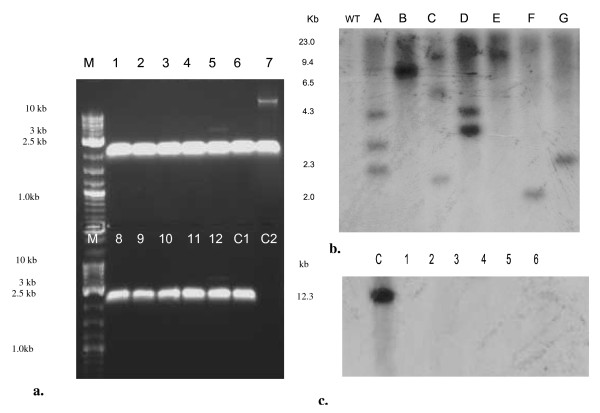
**Molecular screening of transformants**. (a). PCR of transformed mycelia using GUS-GFP fusion primer. Lane M: DNA molecular weight marker (kb); Lane 1&2: *Ganoderma *sp. RCKK-02; Lane 3&4: *P. cinnabarinus*; Lane 5&6: *Crinipellis *sp. RCK-1; Lane 7&8: *P. sojur-caju*; Lane 9&10: *P. chrysosporium*; Lane 11&12: Fungal isolate BHR-UDSC. Lanes 2,4,6,8,10 & 12 are acetosyringone (AS) pre-induced, whereas others are without AS. The Lane C1 & Lane C2 were positive control and negative control, respectively. (b). Southern blot analysis of transformed fungus using radiolabelled *hpt *gene probe. Lane WT: Untransformed; Lane A: *Ganoderma *sp. RCKK-02; Lane B: *P. cinnabarinus*; Lane C: *Crinipellis *sp. RCK-1; Lane D: *P. sojur-caju*; Lane E: Positive control; Lane F: *P. chrysosporium*; Lane G: Fungal isolate BHR-UDSC. (c). Southern blot analysis of different transformants using *Kan^R ^*probe. Lane C: Untransformed control; Lane 1: *Ganoderma *sp. RCKK-02; Lane 2: *P. cinnabarinus*; Lane 3: *Crinipellis *sp. RCK-1; Lane 4: *P. sojur-caju*; Lane 5: *P. chrysosporium; *Lane 6: Fungal isolate BHR-UDSC.

The Hyg^r ^transformants were PCR screened with *gus*-*gfp *primers which resulted in an expected amplified product of 2.5 kb (Figure 5a). Randomly selected transformants were further used for molecular analysis. The *Bam*HI digested genomic DNA from transformants was hybridized to P^32 ^α dATP labelled *htp*. The Southern analysis confirmed transformation and also revealed that the number of inserts in different transformed lines varied from one to four (Figure 5b). All the Hyg^r ^mycelia were tested for the possibility of *Agrobacterium *contamination. Fungal mycelia were grown on LB medium to screen any bacterial contamination. The *Bam*HI digested genomic DNA samples from the transformed lines were analysed by Southern hybridization using *Kan^R ^*probe. No hybridization was detected in the transformants, which ruled out the possibility of any bacterial contamination (Figure 5c).

### Agrobacterium-fungal attachment confirmation

Attachment of *Agrobacterium *to fungal mycelia was confirmed by TEM analysis. The TEM results revealed bacterial attachment to fungal cells. This method of co-culturing might have resulted in a substantial increase in concentration of bacteria which eventually facilitated transfer of T-DNA to fungal cells without wound formation [31,32].

The competence of plant cells for *Agrobacterium *mediated DNA transfer is not necessarily linked to cell damage. T-DNA integration, therefore, does not absolutely need the wounding activities in the plant cell. This indicates that the well-known requisite of a wound for transformation is probably a special sensory attraction that *Agrobacterium *developed to recognize a natural niche [33]. The transfer of T-DNA from *A. tumefaciens *to plant genome, by a type IV secretion system (T4SS), most probably resembles DNA transfer between bacteria during conjugation. Indeed, this transfer mechanism was found to be functional during conjugative transfer of Ti plasmids between *Agrobacterium *and other bacteria as well as plant cells, which in turn suggested that *Agrobacterium *can transfer genetic material with other non-plant species [10]. The *Agrobacterium radiobacter *has been reported to be associated with 10 different strains of *P. chrysosporium *[34]. Thus, it is likely that bacteria and fungi act together either simultaneously or consecutively to degrade lignin or its breakdown products. As *Agrobacterium *and white-rot fungi share a common habitat, they encounter each other very often. Our transformation experiment suggests that T-DNA transfer from *Agrobacterium *to white-rot fungi does indeed occur in nature, and horizontal DNA transfer between kingdoms may be more frequent and extensive, which also support our previous observations [11]. Moreover, basidiomycetes are more closely related to higher eukaryotes, therefore, it is a better choice as a heterologous system for the expression of higher eukaryotic genes.

## Conclusion

The transformation results suggest that co-cultivation at low temperature could significantly increase the transformation frequency. The strategy to use both CaMV 35 S and GPD promoter demonstrated comparable efficacy in the expression of *hpt *and *uidA *genes in white-rot fungi, respectively. Moreover, these findings provide a rapid and reproducible transformation method without external addition of acetosyringone, which could be useful for improving white-rot fungi for their various biotechnological applications.

## Abbreviations

CaMV: cauliflower mosaic virus; MCS: multiple cloning site; GDP: glyceraldehyde 3-phosphate dehydrogenase; MEA: malt extract agar; YEA: yeast extract agar; TEM: transmission electron microscope.

## Authors' contributions

KKS conducted the experiments, analyzed the data and wrote the manuscript. RCK (corresponding author) planned the work, coordinated the study and critically finalised the manuscript. Both agree to submit the manuscript to BMC Biotechnology.
